# Multi-Scale Synergistic Regulation Strategy to Develop Mesoporous Carbon Hollow Nanospheres/Bean-Shaped Nanofibers for Corrosion-Resistant, Flexible, and Lightweight Microwave Absorbers

**DOI:** 10.34133/research.1051

**Published:** 2026-01-15

**Authors:** Hemin Wang, Beibei Zhan, Yiru Zhang, Zhiyun Tan, Junfei Ding, Yanli Chen, Yunpeng Qu, Xiaosi Qi

**Affiliations:** ^1^ College of Physics, Guizhou University, Guiyang 550025, People’s Republic of China.; ^2^ School of Physics and Electronic Science, Zunyi Normal College, Zunyi 563006, People’s Republic of China.

## Abstract

Addressing the critical demand for next-generation lightweight, high-efficiency microwave absorbers, this paper proposes a “micro-meso-macro” multi-scale synergistic regulation strategy. Specifically, core@shell mesoporous carbon hollow nanospheres (HNSs)@carbon bean-shaped nanofibers (BNFs) are designed and fabricated efficiently using SiO_2_/carbon solid nanospheres as precursor through a continuous electrostatic spinning, heat treatment, carbonization, and hydrofluoric (HF) etching. The acquired results suggest that the regulation of carbonization temperature greatly improves the graphitized degree of mesoporous carbon HNSs@carbon BNFs, which significantly enhances the values of complex permittivity. Furthermore, the introduction of a controllable number of mesoporous carbon HNSs at the mesoscale significantly increases the specific surface area and promotes the interfacial polarization effects. The macroscopic 3-dimensional continuous conductive network constructed via electrospinning further enhances electron transport capability and conductive loss efficiency. Benefiting from the excellent collaborative design between multi-scale structure and composition, the optimized mesoporous carbon HNSs@carbon BNFs display excellent microwave absorption properties with a minimum reflection loss (RL_min_) of −61.03 dB at 2.42 mm and an effective absorption bandwidth (EAB) of 6.2 GHz at 2.18 mm. Meanwhile, the acquired mesoporous carbon HNSs@carbon BNFs also present excellent corrosion resistance, hydrophobicity, flexibility, and lightweightness. Generally, the finding proposes a simple route for the production of novel core@shell C@C nanocomposites, which makes the best of multi-scale construction strategy to develop lightweight multifunctional microwave absorbers.

## Introduction

With the rapid advancement of 5G, the Internet of Things, and stealth technology, electromagnetic (EM) pollution and interference have become increasingly severe, posing significant threats to equipment safety, information confidentiality, and human health [1–4]. Consequently, the development of lightweight, broadband, and robust high-performance microwave absorbers (MAs) has emerged as a strategic imperative and urgent research priority within the field of functional materials [5–8]. Therefore, carbon-based nanocomposites (CBNCs) are regarded as desirable candidates for exploiting high-performance MAs due to the unique structures and excellent physical and chemical properties of carbon materials [9–11]. For instance, Che and co-workers [12] employed a 2-step oxidation–reduction process involving chemical etching followed by high-temperature reduction to transform cobalt boron imidazolate framework (Co-BIF) into a 2-dimensional (2D) CoNi alloy embedded within a B,N co-doped carbon layer (CNC). This approach enhanced microwave attenuation performances (MAPs) by simultaneously strengthening the synergistic loss arising from interface/dipole/defect polarization and magnetic coupling. Gu’s team [13] synthesized high aspect ratio chain-like CoNi via polyvinylpyrrolidone (PVP)-directed sol–gel reduction and thermally pressed it with polydimethylsiloxane (PDMS) to fabricate an 18 vol % chain-like CoNi/PDMS flexible carbon–magnetic composite membrane. This composite achieved low-frequency absorption with a minimum reflection loss (RL_min_) of −56.7 dB and absorption bandwidth of 1.04 GHz. Lu and his team [14] fabricated 3D carbon-scaffolded necklace-like Fe_3_O_4_ nanostructures through in situ growth-vacuum filtration–self-reduction calcination process. Boosted impedance matching and interfacial polarization could be fulfilled by modulating their compositions. The optimized CBNCs achieved an RL_min_ of −59.3 dB and an effective absorption bandwidth (EAB) of 5.6 GHz at a thickness of 2.2 mm. Summarizing the progress made earlier, the previous research predominantly focused on the design and development of metal/carbon and/or metal oxide/carbon CBNCs, which actually presented very excellent MAPs. Although the introduction of metal and/or metal oxide nanoparticles effectively improves their MAPs, it also brings many disadvantages such as increased density and degraded environmental stability. Consequently, a lot of research work is still needed to develop CBNCs as lightweight high-performance MAs.

Carbon/carbon CBNCs have received a lot of attention in recent years owing to their lightweight properties, low densities, designable microstructures, and excellent chemical stabilities [15–17]. For example, Zhang’s team [18] employed a vacuum-assisted filtration technique to assemble carbon nanospheres with cellulose nanofibers for constructing lightweight, flexible carbon nanosphere/cellulose nanofiber CBNCs. The synergistic interaction between the 2 components and the formed abundant interfaces endowed the carbon nanosphere/cellulose nanofiber CBNCs with excellent thermal camouflage, low density, and mechanical and EM interference shielding stability. Ozin and co-workers [19] fabricated nitrogen-doped porous carbon (NPC)/carbon nanotube (CNT) CBNCs through a continuous process of solution casting, ammonia cross-linking, and high-temperature carbonization. The unique composition and structure enabled NPC/CNTs CBNCs as desirable gas diffusion electrode, achieving an 81% Faradaic efficiency for CO₂ reduction to formic acid in 0.1 M KHCO₃ stable operation for 36 h. Additionally, Zhang et al. [20] adopted sugar-derived strategy to produce carbon/carbon CBNCs consisting of carbon fiber and reinforced carbon matrix. The designed C/C CBNCs presented excellent properties including thermal, mechanical, and ablation resistance owing to a unique interfacial transition zone and a strong bond formed between sugar-derived graphite and carbon fiber. In summary, carbon/carbon CBNCs are demonstrated to display outstanding advantages in weight reduction, corrosion resistance, thermochemical stability, service life, and so on [21–24]. Continuously pushing the boundaries of performance in lightweight, high stability, and mechanical flexibility, it has become a universal platform for cross-scale, cross-domain functional integration. Designing the customizable carbon/carbon CBNCs to take full advantage of interface engineering is a desirable strategy to develop lightweight high-performance MAs owing to their superior environmental adaptabilities and overall performances.

Based on previous work [25], herein, a novel core@shell structured carbon@carbon CBNCs comprising mesoporous carbon hollow nanospheres (HNSs) and carbon nanofibers (CNFs) were elaborately designed and produced in high efficiency through a sequential process of electrospinning, carbonization, and hydrofluoric (HF) etching. The crystallinity of mesoporous carbon HNSs@carbon bean-shaped nanofibers (BNFs) could be effectively regulated at the microscopic level by controlling the carbonization temperature to achieve the programmable dielectric constants. Mesoporous carbon HNSs were quantitatively incorporated into designed mesoporous carbon HNSs@carbon BNFs to establish mesoscale gradient interface polarization. Furthermore, at the macroscale, electrospinning weaved fibers into a 3D continuous conductive network, balancing electron transport with multiple scattering effects. Therefore, a multi-scale collaborative strategy was proposed and employed to develop mesoporous carbon HNSs@carbon BNFs with excellent corrosion resistance, hydrophobicity, flexibility, lightweight, and MAPs.

## Results and Discussion

**Fig. 1.**
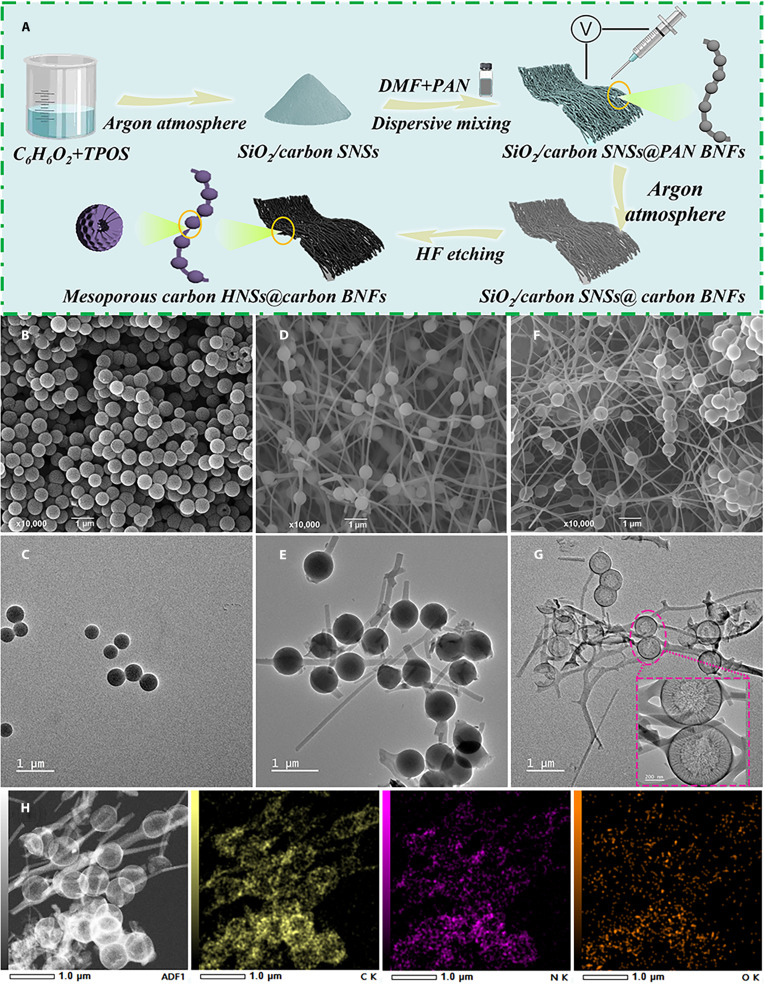
(A) Synthesis flowchart of mesoporous carbon HNSs@carbon BNFs. SEM and TEM of (B and C) SiO_2_/carbon SNSs, (D and E) SiO_2_/carbon SNSs@PAN BNFs, and (F and G) CCBNFs-1. (H) TEM element mapping images of CCBNFs-1.

Figure 1 presents the synthesis flowchart and microstructures of SiO_2_/carbon solid nanospheres (SNSs), SiO_2_/carbon SNSs@PAN (polyacrylonitrile) BNFs, and mesoporous carbon HNSs@carbon BNFs. As shown in Fig. 1A, SiO_2_/carbon SNSs were firstly produced via a straightforward polymerization reaction and carbonization process using C_6_H_6_O_2_ and tetrapropoxysilane (TPOS) as carbon and silicon sources, respectively. Subsequently, the resulting SiO_2_/carbon SNSs were uniformly dispersed with *N*,*N*-dimethylformamide (DMF)/PAN to produce SiO_2_/carbon SNSs@PAN BNFs through the electrostatic spinning process. After the continuous heat treatment and carbonization process, the generated PAN was converted to carbon, which resulted in the formation of SiO_2_/carbon SNSs@carbon BNFs. Finally, mesoporous carbon HNSs@carbon BNFs were successfully fabricated in high efficiency after the removal of SiO_2_ from SiO_2_/carbon SNSs by HF etching. As shown in Fig. 1B and C, the scanning electron microscopy (SEM) and transmission electron microscopy (TEM) images suggest that the acquired SiO_2_/carbon SNSs show a typical solid spherical morphology with uniform sizes (Fig. S1A and B, average diameter: 492.9 nm) and smooth surfaces, indicating the large-scale production of SiO_2_/carbon SNSs. From the SEM observation (Fig. 1D), the outcome suggests that a typical morphology of BNFs consisting of nanospheres and nanofiber can be observed over the acquired SiO_2_/carbon SNSs@PAN BNFs. The TEM image (Fig. 1E) further confirms that a certain amount of SiO_2_/carbon SNSs acting as core is embedded in the shell PAN nanofiber matrix, which shows a clear core@shell structure. The incorporation of fibers enables the construction of continuous 3D conductive pathways, significantly enhancing electronic transport efficiency. After the continuous carbonization and HF etching process, the SEM investigation (Fig. 1F) reveals that CCBNFs-1 still retains the morphology of BNFs, which is composed of nanospheres and nanofiber. The TEM images (Fig. 1G and inset) demonstrate that the generated nanospheres encapsulated in the PAN nanofiber display an evident mesoporous and hollow structures, indicating the formation of mesoporous carbon HNSs@carbon BNFs [26]. Similar to those of CCBNFs-1, the SEM and TEM images also confirm that the obtained CCBNFs-2 (Fig. S1C and D) and CCBNFs-3 (Fig. S1E and F) consist of mesoporous carbon HNSs and carbon BNFs. This unique mesoporous and hollow structure facilitates multiple reflections and scattering, while mesoporous interfaces induce strong polarization loss to achieve excellent impedance matching characteristics and lightweight properties [27]. In addition, the typical TEM elemental mapping diagrams of CCBNFs-1 (Fig. 1H) also confirm the mesoporous and hollow structures of inner carbon nanospheres and BNFs of mesoporous carbon HNSs@carbon, which is composed of C, N, and O elements. Overall, SEM and TEM results demonstrate that mesoporous carbon HNSs@carbon BNFs with 3D conductive network are successfully synthesized in high efficiency through the proposed route.

**Fig. 2.**
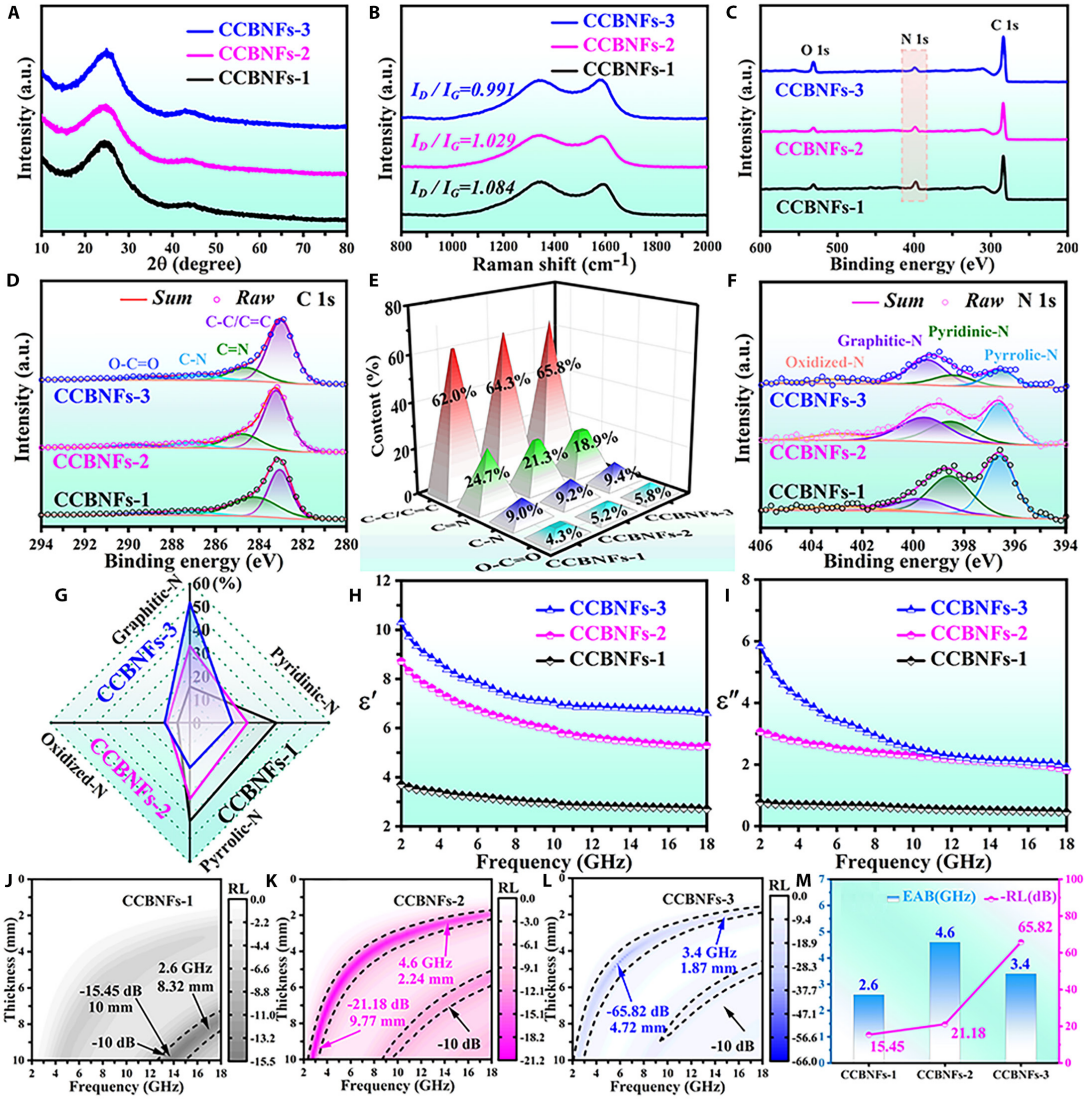
(A to G) XRD, Raman, and XPS spectra. (H and I) Complex permittivity. (J to M) RL and EAB values of CCBNFs-1, CCBNFs-2, and CCBNFs-3.

Figure 2 provides the x-ray diffraction (XRD), Raman and x-ray photoelectron spectroscopy (XPS) spectra, complex permittivity, and reflection loss (RL) results of CCBNFs-1, CCBNFs-2, and CCBNFs-3. As shown in Fig. 2A, CCBNFs-1, CCBNFs-2, and CCBNFs-3 exhibit the (002) and (100) characteristic diffraction peaks corresponding to carbon [28]. With the carbonization temperature increasing from 700 to 900 °C, the (002) peak position gradually shifts from 24.4° to 24.9°. The shift of peak position toward the positive direction indicates that elevated temperature promotes the transformation of amorphous carbon toward graphitization [29]. The characteristic peaks (Fig. 2B) at ca. 1,348 cm^−1^ (D peak) representing the disordered carbon structure and 1,582 cm^−1^ (G peak) corresponding to graphitized carbon are observed over CCBNFs-1, CCBNFs-2, and CCBNFs-3. The $I_{D}/I_{G}$ ratio gradually decreases from 1.08 (CCBNFs-1) to 1.03 (CCBNFs-2) and 0.99 (CCBNFs-3), which further confirm the gradual enhancement of graphitized degree for obtained samples [30]. To further determine the chemical composition, XPS characterization was performed. As shown in Fig. 2C, all the acquired mesoporous carbon HNSs@carbon BNFs show the obvious C 1s, N 1s, and O 1s characteristic peaks. It is worth noting that the intensity of N 1s peak for mesoporous carbon HNSs@carbon BNFs gradually decreases by increasing the carbonization temperature, indicating a decreased trend of N content caused by the volatilization or decomposition of N under high-temperature conditions [31]. In addition, the high-resolution XPS spectra of C 1s (Fig. 2D) can be fitted by C–C/C=C (283.2 eV), C=N (284.5 eV), C–N (284.9 eV), and O–C=O (288.8 eV) bonds [32,33]. As given in Fig. 2E, the C–C/C=C content in the obtained sample is also gradually increased from CCBNFs-1 to CCBNFs-2 and CCBNFs-3, which is also consistent with the XRD and Raman results. The enhancement of graphitization favorably contributes to the enhancement of electrical conductivity [34]. Additionally, the obtained mesoporous carbon HNSs@carbon BNFs present the decreased C=N bond content and a slight enhanced C–N and O–C=O bond contents, which is due to the partial breakage and rearrangement of C=N bond at high temperatures. This facilitates the enhancement of structural ordering and manifests as a slight rightward shift in the overall peak position [35]. As provided in Fig. 2F, the high-resolution N 1s XPS spectra of CCBNFs-1, CCBNFs-2, and CCBNFs-3 show a decreasing trend in N content. The N 1s spectra are deconvoluted into 4 typical nitrogen configurations: pyrrole nitrogen (~396.7 eV), pyridine nitrogen (~397.9 eV), graphitic nitrogen (~399.3 eV), and nitrogen oxide (402.0 eV) [36]. The quantitative analysis results (Fig. 2G) show that the obtained mesoporous carbon HNSs@carbon BNFs present gradually increased contents of graphite nitrogen and nitrogen oxide by increasing the carbonization temperature. According to the previous results [37], the enhancement of graphitic nitrogen can enhance the *sp^2^* conjugated network of carbon skeleton, which is conducive to optimize the electrical conductivity. In summary, XRD, Raman, and XPS results indicate that precise temperature control enables microscopic regulation of nitrogen doping concentration and crystallinity. As shown in Fig. 2H and I, owing to the intrinsic properties of carbon materials and their hollow structures, the obtained fibers exhibit high complex permittivity values even at a low filling ratio of 12.5%. The complex permittivity ($\varepsilon =\varepsilon^{\prime }-i\varepsilon^{\prime \prime }$) values of CCBNFs-1, CCBNFs-2, and CCBNFs-3 show a decreasing trend within 2 to 18 GHz, which are in agreement with the Debye theory. In addition, both $\varepsilon^{\prime }$ (Fig. 2H) and $\varepsilon^{\prime \prime }$ (Fig. 2I) show a pattern of CCBNFs-1 < CCBNFs-2 < CCBNFs-3, which implies the enhanced electrical conductivity [38]. Compared with mesoporous carbon HNSs@carbon BNFs, the SiO_2_/carbon SNSs@PAN BNFs exhibit the relatively low $\varepsilon^{\prime }$ and $\varepsilon^{\prime \prime }$ values (Fig. S2A) owing to the presence of silica and solid structure. The obtained complex permittivity results are consistent with the XRD, Raman, and XPS findings. As shown in the 2D RL diagrams for CCBNFs-1 (Fig. 2J), CCBNFs-2 (Fig. 2K), CCBNFs-3 (Fig. 2L), and SiO₂/carbon SNSs@PAN BNFs (Fig. S2B), CCBNFs-2 exhibits a superior EAB value. To further investigate variations in its MAPs, Fig. 2M provides the 2D RL plots of CCBNFs-1, CCBNFs-2, and CCBNFs-3, which have an RL_min_ value of −15.45 dB at 13.6 GHz, −21.18 dB at 2.8 GHz, and −65.82 dB at 5.8 GHz. Their corresponding *d*_m_ values of RL_min_ values are 10.00, 9.77, and 4.72 mm. Furthermore, the EAB values of CCBNFs-1 (Fig. 2J), CCBNFs-2 (Fig. 2K), and CCBNFs-3 (Fig. 2L) are 2.60 GHz at 8.32 mm (15.4 to 18 GHz), 4.60 GHz at 2.24 mm (13.2 to 17.8 GHz), and 3.40 GHz at 1.87 mm (14.6 to 18 GHz), respectively.

**Fig. 3.**
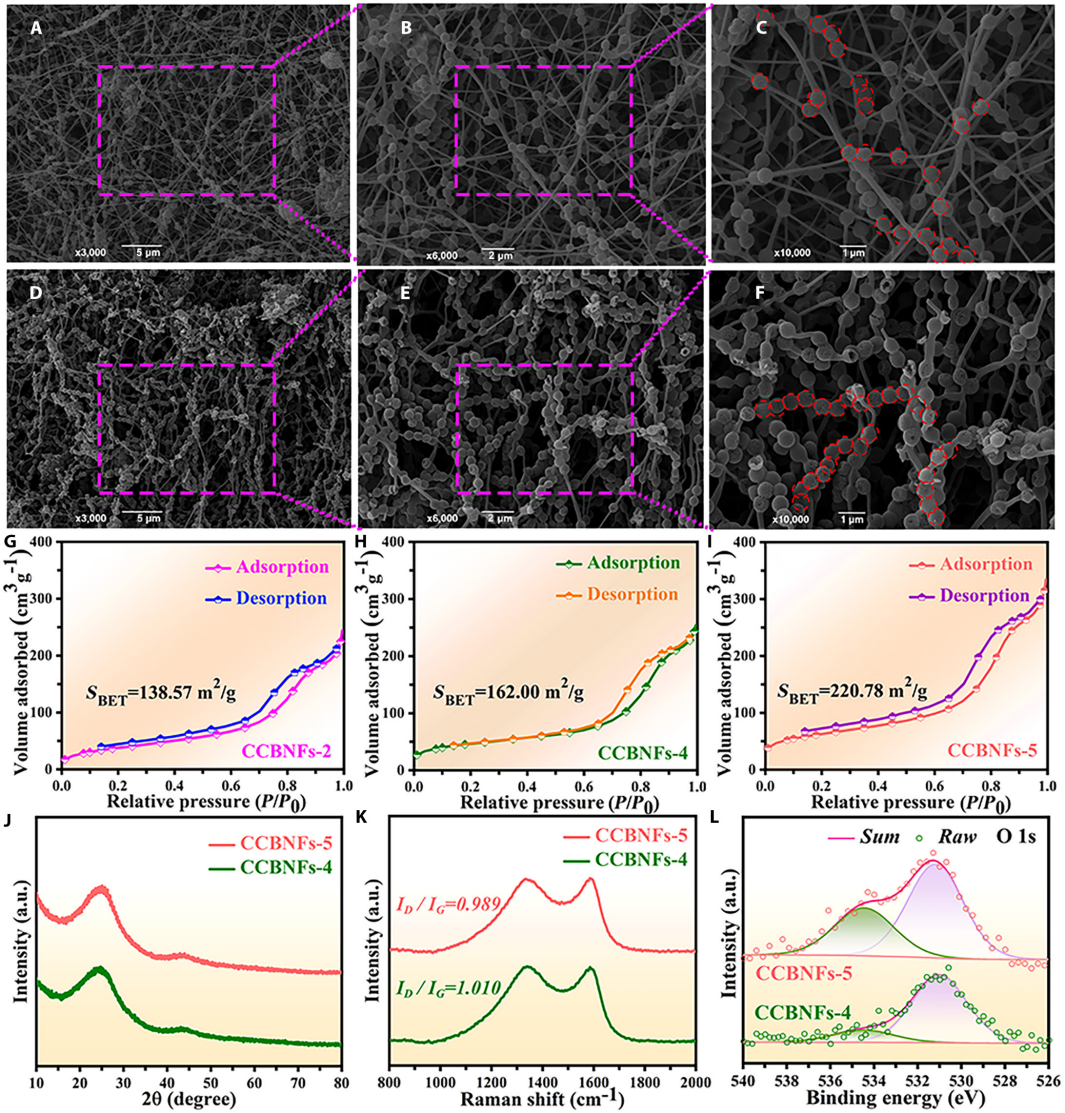
(A to F) SEM images, (G to I) BET, and (J to L) XRD, Raman, and XPS spectra of CCBNFs-4 and CCBNFs-5.

To further optimize the EM parameters and MAPs, this study incorporated more mesoporous carbon HNSs at the mesoscale. Following the design and fabrication described in Materials and Methods, the obtained mesoporous carbon HNSs@carbon BNFs are labeled as CCBNFs-4 and CCBNFs-5. As shown in Fig. 3A to C, the SEM observation reveals that the acquired CCBNFs-4 shows a typical neural network-like nanofiber consisting of mesoporous HNSs (as marked by the circles in Fig. 3C) and nanofiber. Equally, similar to CCBNFs-2 and CCBNFs-4, the SEM investigation (Fig. 3D to F) also confirms the typical BNF morphology of CCBNFs-5. The comparison results of SEM images evidently confirm that much more quantity of mesoporous carbon HNSs (as labeled by the circles in Fig. 3F) acting as core are observed into the acquired mesoporous carbon HNSs@carbon BNFs from CCBNFs-2 to CCBNFs-4 and CCBNFs-5, which originates from the enhanced introduction of SiO_2_/carbon SNSs. Additionally, the TEM and elemental mapping images of CCBNFs-4 (Fig. S2C and D) and CCBNFs-5 (Fig. S2E and F) demonstrate their same hollow structures and elemental compositions as CCBNFs-2. As provided in Fig. 3G to I, the specific surface areas of CCBNFs-2, CCBNFs-4, and CCBNFs-5 increase evidently from 138.57 m^2^ g^−1^ to 162.00 and 220.78 m^2^ g^−1^, respectively. One can find that the progressive number of mesoporous carbon HNSs significantly enhances the amounts of surface activity. The enlarged specific surface area promotes interfacial polarization, multiple scattering, and defect losses, thereby substantially improving the MAPs of the material [39]. The XRD spectra (Fig. 3J) confirm that both CCBNFs-4 and CCBNFs-5 display the characteristic peaks corresponding to carbon at 24.7° (002 crystal plane) and 43.6° (100 crystal plane) [40]. Raman spectroscopy analysis (Fig. 3K) shows that the $I_{D}/I_{G}$ values of CCBNFs-4 (1.01) and CCBNFs-5 (0.99) are further reduced compared to that of CCBNFs-2 (1.03), which indicates an increased degree of graphitization [41,42]. Additionally, the XPS full spectrum analysis (Fig. S3A) also confirms the existence of C, N, and O elements over the acquired CCBNFs-4 and CCBNFs-5. As shown in Fig. 3L and Fig. S3B, the XPS high-resolution spectra of O 1s for CCBNFs-2, CCBNFs-4, and CCBNFs-5 show the presence of defective and adsorbed oxygen at 531 and 535 eV. The contents of adsorbed oxygen and defective oxygen (Fig. S3C) for CCBNFs-2, CCBNFs-4, and CCBNFs-5 are 14.68%, 16.65%, and 35.48%, and 85.32%, 83.35%, and 64.52%, respectively. The increased adsorbed oxygen and decreased defective oxygen contents are attributed to the increased specific surface area of mesoporous carbon HNSs@carbon BNFs with the enhanced introduction of mesoporous carbon HNSs [43], which is consistent with the results of Brunauer–Emmett–Teller (BET). Similarly, the XPS high-resolution spectra of N 1s (Fig. S3D) show the presence of 4 diffraction peaks of pyrrole nitrogen (~396.7 eV), pyridine nitrogen (~397.9 eV), graphitic nitrogen (~399.3 eV), and nitrogen oxide (~402 eV). As shown in Fig. S3E, the acquired mesoporous carbon HNSs@carbon BNFs also present the sequentially enhanced content of graphite nitrogen by increasing the addition of mesoporous carbon HNSs. In summary, the collected results demonstrate that mesoporous carbon HNSs@carbon BNFs with different contents of mesoporous carbon HNSs and specific surface areas can be selectively produced by controlling the amount of SiO_2_/carbon SNSs, which provides an effective path to optimize EM and impedance matching properties.

**Fig. 4.**
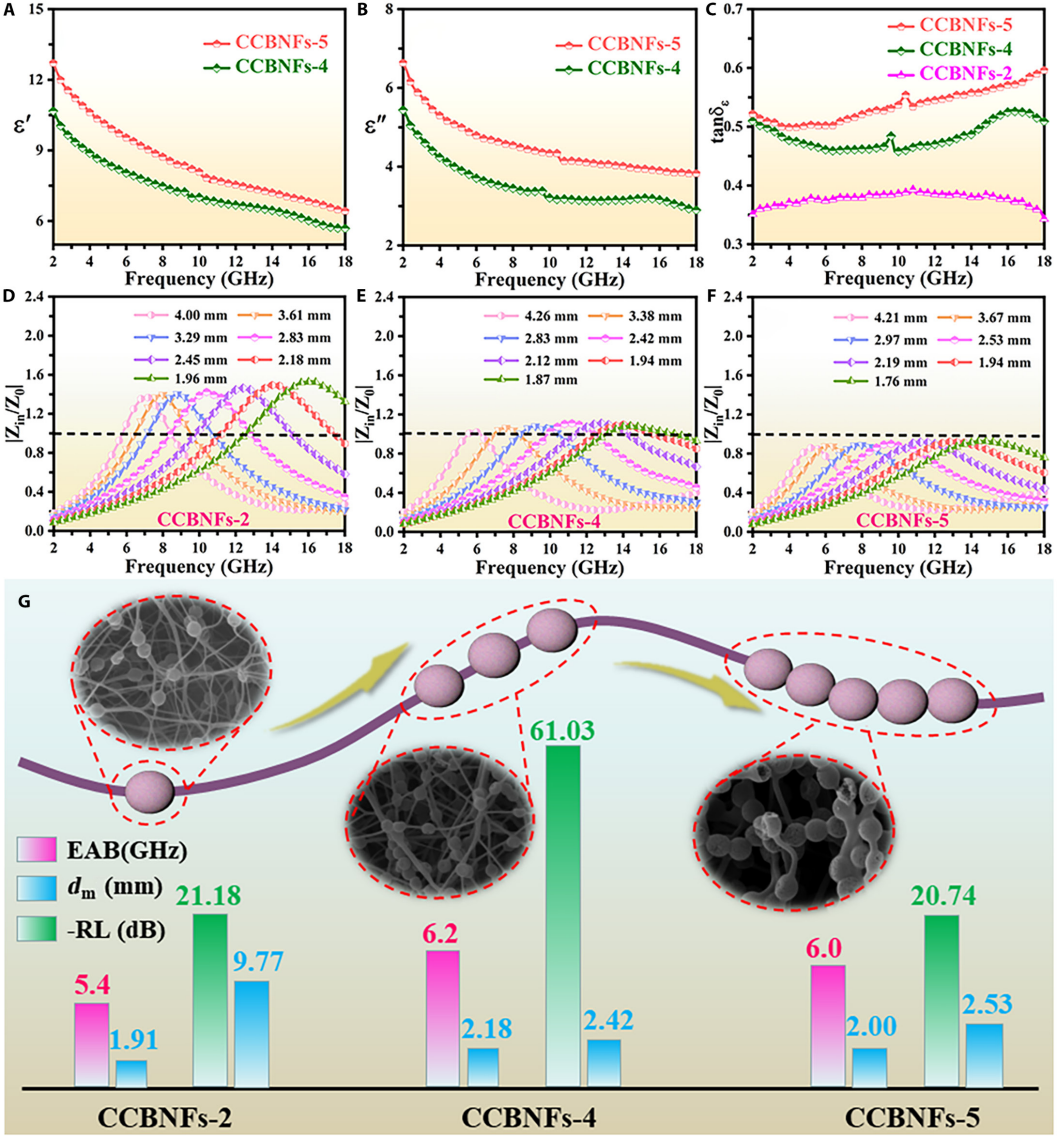
(A and B) Complex permittivity, (C) $\tan\delta_{\varepsilon }$, (D to F) Z, and (G) MAPs of CCBNFs-2, CCBNFs-4, and CCBNFs-5.

To evaluate the MAPs, Fig. 4 provides the complex permittivity, dielectric loss tangent ($\tan\delta_{\varepsilon }=\varepsilon^{\prime \prime }/\varepsilon^{\prime }$), impedance matching, and RL values for CCBNFs-4 and CCBNFs-5. As shown in Fig. 4A, the $\varepsilon^{\prime }$ values of CCBNFs-4 and CCBNFs-5 locate between 10.68 and 5.69, 12.71, and 6.42. Similarly, their $\varepsilon^{\prime \prime }$ values (Fig. 4B) vary between 5.44 and 2.90 and 6.34 and 3.83. Compared to CCBNFs-2, CCBNFs-4 and CCBNFs-5 exhibit a significant increase of $\varepsilon^{\prime }$ and $\varepsilon^{\prime \prime }$ values, following the pattern CCBNFs-2 < CCBNFs-4 < CCBNFs-5. The comparative results suggest that the increased addition of mesoporous carbon HNSs significantly boosts the value of complex permittivity for mesoporous carbon HNSs@carbon BNFs, which is very beneficial for enhancing the MAPs [44,45]. Additionally, the $\tan\delta_{\varepsilon }$ values (Fig. 4C) for mesoporous carbon HNSs@carbon BNFs are as follows: CCBNFs-2 < CCBNFs-4 < CCBNFs-5, implying the boosted dielectric loss capabilities [46]. Based on the acquired outcomes, the enhanced introduction of mesoporous carbon HNSs greatly strengthens the specific surface area and creates additional pathways for electron transport, thereby heightening dielectric loss abilities [47]. To understand the penetration of EM waves, Fig. 4D to F presents the $Z=\left|Z_{\text{in}}/Z_{0}\right|$ values to evaluate the impedance matching performances of mesoporous carbon HNSs@carbon BNFs. The contrastive results demonstrate that the Z values of CCBNFs-4 and CCBNFs-5 are close to 1 compared with CCBNFs-2, implying the improved optimization of impedance matching performances by enhanced introducing mesoporous carbon HNSs [48,49]. CCBNFs-4 displays the best impedance matching characteristic. As shown in Fig. S4A to C, CCBNFs-4 and CCBNFs-5 exhibit the RL_min_ value of −61.03 dB at 2.42 mm and −20.74 dB at 2.53 mm, respectively. Additionally, the EAB values of CCBNFs-4 and CCBNFs-5 are 6.2 GHz (11.8 to 18 GHz) at 2.18 mm and 6.0 GHz (12 to 18 GHz) at 2.00 mm. As summarized in Fig. 4G, the obtained CCBNFs-4 and CCBNFs-5 present much lower RL_min_, larger EAB, and smaller *d*_m_ values than CCBNFs-2, which demonstrates that increased addition of mesoporous carbon HNSs significantly improves the comprehensive MAPs of mesoporous carbon HNSs@carbon BNFs. The obtained CCBNFs-4 displays the superior comprehensive MAPs compared with CCBNFs-5, which should be ascribed to the slightly degraded impedance matching performances induced by the excessive introduction of mesoporous carbon HNSs. In summary, the comprehensive MAPs of designed mesoporous carbon HNSs@carbon BNFs can be significantly improved by controlling the introduction of mesoporous carbon HNSs. More importantly, compared with other representative CBNCs (Table 1), the optimized mesoporous carbon HNSs@carbon BNFs combine the advantages of low density, strong attenuation, broad absorption bandwidth, and thin matching thickness, which mainly originates from the excellent synergistic effect between unique structure and composition.

**Table 1.** Comparison table about MAPs with the recently reported representative works

Materials	EAB (GHz)	Thickness (mm)	RL_**min**_ (dB)	Thickness (mm)	References
RGO/MWCNTs	3.34	1.50	−57.36	2.90	[5]
MoS_2_@C	3.70	2.63	−62.30	2.88	[6]
BN_7.5_@Co-C@C/PET	1.28	4.80	−63.10	4.80	[10]
Fe_3_O_4_-Fe@CNFs	5.60	2.20	−59.30	4.30	[15]
NHCS@NiO/Ni	4.38	1.70	−44.04	2.00	[27]
CoNi-LDH	4.30	2.20	−80.00	3.20	[34]
CNF-PN	6.60	2.30	−55.00	2.30	[37]
Carbon fiber	7.00	2.50	−39.90	3.00	[40]
MCHS	5.40	3.20	−50.90	3.20	[41]
SiC/C	5.10	1.80	−60.80	2.70	[44]
CCBNFs-4	6.20	2.18	−61.03	2.42	This work
CCBNFs-5	6.00	2.00	−20.74	2.53	This work

**Fig. 5.**
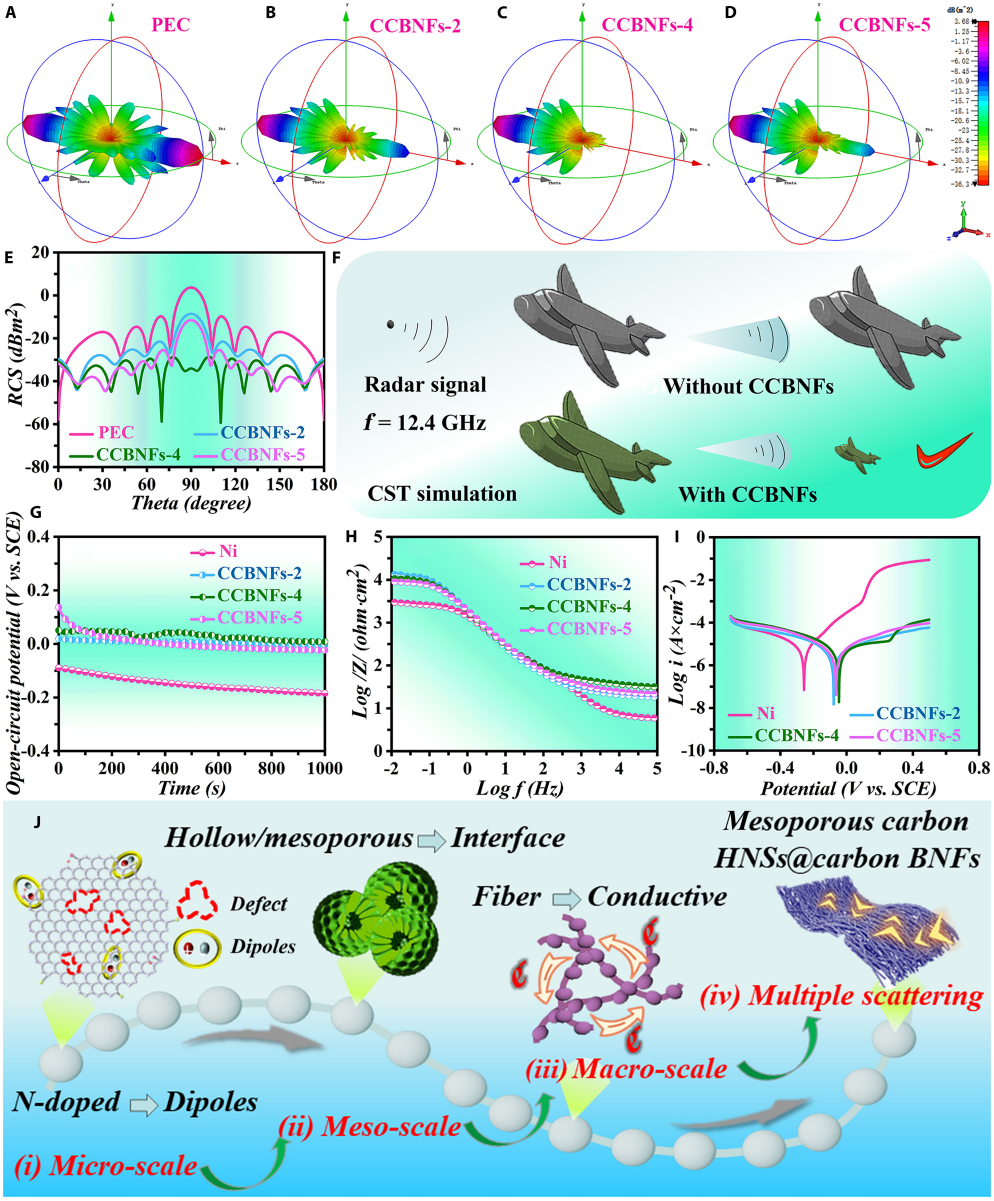
(A to F) RCS simulation. (G to I) Open-circuit, bode plots, and Tafel curves. (J) EM wave absorption mechanism of mesoporous carbon HNSs@carbon BNFs.

To further evaluate the practical MAPs of mesoporous carbon HNSs@carbon BNFs, radar cross-section (RCS) simulations of coated samples were conducted using CST Microwave Studio software [50,51]. The results suggest that the pure perfect electric conductor (PEC) plate (Fig. 5A) exhibits the near-total reflection of radar signals. In contrast, all the plates coated with CCBNFs-2 (Fig. 5B), CCBNFs-4 (Fig. 5C), and CCBNFs-5 (Fig. 5D) significantly attenuate the radar scattering signal. Among these plates, the plate coated with CCBNFs-4 displays the weakest scattering signal, indicating its optimal MAPs. The RCS curves (Fig. 5E) reveal that all the plates coated with mesoporous carbon HNSs@carbon BNFs display much lower RCS values than pure PEC plate across the whole detection angle range. Notably, the RCS value of CCBNFs-4 remains almost entirely below −30 dBm^2^, indicating extremely weak reflection of incident radar waves and meeting the performance requirements for stealth materials [52,53]. The computer simulation technology (CST) simulation schematic (Fig. 5F) further demonstrates that at 12.4 GHz, an uncoated target remains clearly detectable by radar, whereas the target coated with the designed mesoporous carbon HNSs@carbon BNFs exhibits low observability. These simulation results collectively confirm that the fabricated mesoporous carbon HNSs@carbon BNFs possess a considerable potential for stealth applications [54,55]. Furthermore, to evaluate suitability, the corrosion resistance of mesoporous carbon HNSs@carbon BNFs was assessed using electrochemical methods [56]. As shown in Fig. 5G to I, the results of open-circuit potential measurements, bode plots, and Tafel curves collectively demonstrate that the designed mesoporous carbon HNSs@carbon BNFs exhibit the outstanding corrosion resistance in simulated seawater solutions, which is primarily attributed to the inherent chemical stability of carbon material itself [57]. Based on the collected results [58,59], the main EM wave attenuation mechanisms of mesoporous carbon HNSs@carbon BNFs (Fig. 5J) primarily encompass the following aspects: (a) N-doping and mesoporous carbon HNS structures introduce additional defects at the microscopic scale, thereby further enhancing the dipole polarization process [60]. (b) The unique BNFs consisting of mesoporous carbon HNSs and CNFs generate abundant interfaces at the mesoscale, which promotes the surface charge accumulation and thereby strengthens the interfacial polarization effect [61]. (c) At the macroscale, BNFs establishes an ideal 3D conductive network, which significantly enhances the material’s conduction loss properties [62]. (d) The generated BNFs effectively extend the propagation path of EM waves, achieving the efficient dissipation of EM wave through multiple reflections and scattering [63].

**Fig. 6.**
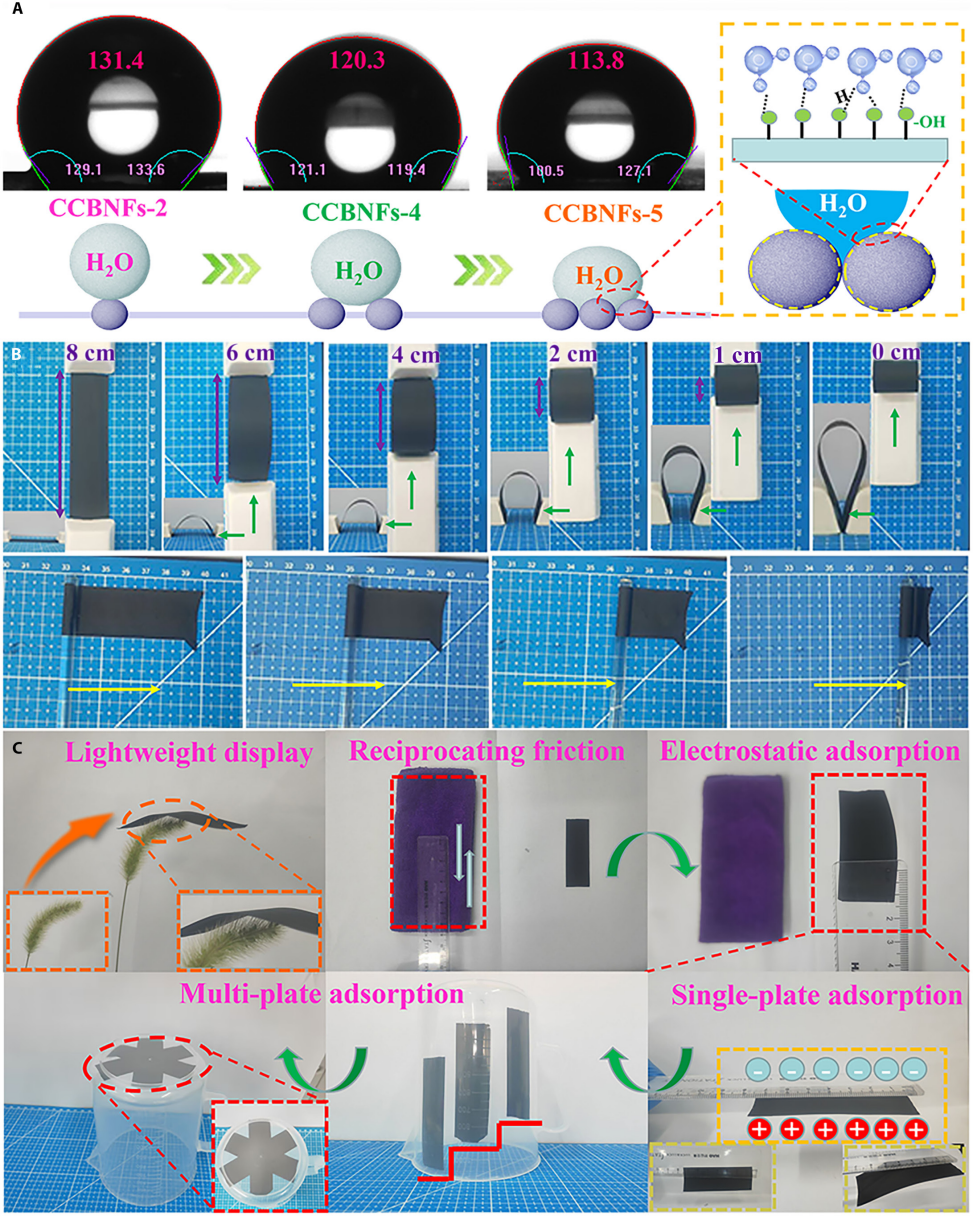
(A) Corrosion resistance. (B) Digital photographs of folded and curled items captured by CCBNFs-4. (C) Lightweight demonstration images of CCBNFs-4.

To further investigate the applicability in practical applications, Fig. 6 provides the results of hydrophobicity, flexibility, and lightweight characteristics of mesoporous carbon HNSs@carbon BNFs. As shown in Fig. 6A, the water contact angles of mesoporous carbon HNSs@carbon BNFs are larger than 110°, indicating their good hydrophobicity and their humidity tolerance [64]. The acquired CCBNFs-2, CCBNFs-4, and CCBNFs-5 exhibit a sequentially decreased water contact angles from 131.4° to 120.3° and 113.8°, indicating a gradual reduction in surface hydrophobicity. According to the classical Wenzel model [65], the increase in micro/mesopores and oxygen-containing functional groups greatly enlarges the actual solid–liquid contact area, macroscopically manifesting as a decrease in contact angle. Taking CCBNFs-4 as an example, as provided in Fig. 6B, 2 porcelain boats were used as bending arms to gradually bend the film of mesoporous carbon HNSs@carbon BNFs with an initial length of 8 cm to 6, 4, 3, 2, 1, and 0 cm. Both the top and front views show that the obtained mesoporous carbon HNSs@carbon BNFs display no fracture and structural damage after slowly rolling it from left to right using a glass rod, which demonstrate that the mesoporous carbon HNSs@carbon BNF film still retains excellent flexibility even after carbonization and etching processes [66,67]. This performance stems from the mesoporous carbon spheres acting as “compressible units” that effectively buffer and disperse stress, while the 3D entangled network enhances energy dissipation capabilities. The synergistic interaction between these 2 mechanisms endows the material with outstanding resistance to bending and fracture [68–70]. To evaluate the density, Fig. 6C provides 2 simple demonstration experiments. The obtained mesoporous carbon HNSs@carbon BNF film can be placed upon blades of grass without causing them to bend. Furthermore, when a negatively charged ruler (rubbed with a towel) is brought close to the acquired mesoporous carbon HNSs@carbon BNF film, the film was rapidly attracted and adhered to the underside of ruler. Using the similar method, multiple pieces of mesoporous carbon HNSs@carbon BNF film are also stably adsorbed onto the sidewall and bottom of a plastic cup. All these phenomena demonstrate the notably lightweight characteristic of designed mesoporous carbon HNSs@carbon BNF film [71,72]. In summary, all the obtained results confirm the excellent hydrophobicity, flexibility, and lightweight characteristics of elaborately constructed mesoporous carbon HNSs@carbon BNFs, which provides an effective strategy for developing multifunctional MAs adaptable to various environments.

## Conclusion

Using SiO_2_/carbon SNSs as precursor, mesoporous carbon HNSs@carbon BNFs can be produced in high efficiency through a continuous process of electrostatic spinning, heat treatment, carbonization process, and HF etching. At the microlevel, the precise control of carbonization temperature enables the effective regulation of fiber crystallinity and nitrogen doping concentration, thereby significantly enhancing the material’s dielectric response and polarization loss capacity. At the mesoscopic level, the controllable incorporation of mesoporous carbon HNSs effectively optimizes their specific surface area and interfacial polarization behavior, which greatly improve their MAPs. At the macroscopic level, the spinning process constructs a 3D continuous conductive network, which endows the mesoporous carbon HNSs@carbon BNFs with excellent flexibility and markedly improved electron transport efficiency. Benefiting from the excellent collaborative design between multi-scale structure and composition, the optimized mesoporous carbon HNSs@carbon BNFs display excellent corrosion resistance, hydrophobicity, flexibility, lightweight, and MAPs with an RL_min_ value of −61.03 dB at 2.42 mm and an EAB value of 6.2 GHz at 2.18 mm, which display a considerable potential for stealth applications. Generally, this research provides an effective multi-scale construction strategy for developing lightweight multifunctional MAs.

## Materials and Methods

### Fabrication of SiO_2_/carbon SNSs

Firstly, SiO_2_/carbon SNSs could be acquired in large quantities according to our previous route [73]. Briefly, 28 ml of deionized water (DI), 140 ml of absolute ethanol, 8 ml of ammonium hydroxide (NH_3_·H_2_O, 28 wt %), 0.8 g of resorcinol, 1 ml of formaldehyde, and 15 ml of TPOS were used as precursors. After a continuous repeated washing with DI, drying overnight at 60 °C, and heat treating at 800 °C for 3 h in Ar, SiO_2_/carbon SNSs could be acquired in large quantities.

### Synthesis of mesoporous carbon HNSs@carbon BNFs

Briefly, mesoporous carbon HNSs@carbon BNFs were produced in high selectivity through a continuous electrostatic spinning, heat treatment, and HF etching. Firstly, 0.5 g of SiO_2_/carbon SNSs and 0.4 g of PAN were dispersed into 5 ml of DMF solution and stirred for 12 h to obtain a mixed solution. Afterward, network-like SiO_2_/carbon SNSs@PAN BNFs could be obtained through the electrostatic spinning treatment of the above solution (operating voltage: 16 kV, needle size: 20-gauge, humidity: 30%). Subsequently, the obtained SiO_2_/carbon SNSs@PAN BNFs were heat treated in air at 260 °C for 2 h and then carbonized at 700 °C under Ar environment for 3 h to obtain SiO_2_/carbon SNSs@carbon BNFs. Finally, mesoporous carbon HNSs@carbon BNFs labeled as CCBNFs-1 were produced in high efficiency by removing SiO_2_ within SiO_2_/carbon SNSs by HF. Similarly, with the other experimental conditions unchangeable, mesoporous carbon HNSs@carbon BNFs labeled as CCBNFs-2 and CCBNFs-3 could also be produced when the carbonization temperatures were conducted at 800 and 900 °C, respectively. Additionally, as summarized in Table 2, mesoporous carbon HNSs@carbon BNFs with different contents of mesoporous carbon HNSs named as CCBNFs-4 and CCBNFs-5 were selectively fabricated using different amounts of SiO_2_/carbon SNSs (0.7 and 0.9 g) as precursors.

**Table 2.** Summarized experimental conditions for the production of mesoporous carbon HNSs@carbon BNFs

Amount of SiO_2_/carbon SNSs (g)	Carbonization temperature (°C)	Abbreviation of sample
0.5	700	CCBNFs-1
0.5	800	CCBNFs-2
0.5	900	CCBNFs-3
0.7	800	CCBNFs-4
0.9	800	CCBNFs-5

## Data Availability

All the data needed to evaluate the conclusions in the paper are present in the paper and in the Supplementary Materials. Additional data related to this paper may be requested from the authors.
