# Supplementary material for: Multi-Scale Synergistic Regulation Strategy to Develop Mesoporous Carbon Hollow Nanospheres/Bean-Shaped Nanofibers for Corrosion-Resistant, Flexible, and Lightweight Microwave Absorbers
Source: Research (Wash D C). 2026 Jan 15;9:1051. doi: 10.34133/research.1051 (PMC12805010; doi:10.34133/research.1051)
Supplement: Supplementary 1 — Supplementary Text Figs. S1 to S4 [file research.1051.f1.doc]

***Supporting Information***

**Multi-Scale Synergistic Regulation Strategy to Develop Mesoporous Carbon Hollow Nanospheres/Bean-Shaped Nanofibers for Corrosion Resistance, Flexible and Lightweight Microwave Absorbers**

Hemin Wang^1,#^, Beibei Zhan^1,#^, Yiru Zhang^1^, Zhiyun Tan^2^, Junfei Ding^1^, Yanli Chen^1^,Yunpeng Qu^1^, and Xiaosi Qi^1*^

^1^College of Physics, Guizhou University, Guiyang 550025, The People’s Republic of China

^2^School of Physics and Electronic Science, Zunyi Normal College, Zunyi 563006, The People’s Republic of China

Correspondence

Xiaosi Qi, College of Physics, Guizhou University, Guiyang 550025, The People’s Republic of China

E-mail: [xsqi@gzu.edu.cn](mailto:xsqi@gzu.edu.cn)

**2. Experimental preparation**

**2.1 Characterization and measurement**

An electrospinning machine (Yong Kang Le Ye Co) and quartz tube furnace (model BTF-1200C-S-SL, Anhui BEQ Equipment Technology CO., LTD) were utilized for material preparation. And X-ray powder diffraction (XRD) (model Smart Lab, Rigaku), electrochemical Raman spectroscopy detection system (EC-Raman), X-ray photoelectron spectroscopy (XPS) (Escalab 250Xi, Thermo Fisher Scientific), scanning electron microscope (SEM) (JIB-4700F, Japan) and transmission electron microscopy (TEM) (JEM-F200, Japan) were used to characterize their phases, chemical compositions and microstructures. Hydrophobic performance evaluations were conducted using an optic surface analyzer (OSA60). The specific surface area was evaluated using a fully automated physical adsorption analyzer (BET, American McASAP 2460). To assess corrosion resistance, the samples were uniformly coated onto nickel sheets and tested using an electrochemical workstation (CHI 760E). A 3.5 wt% sodium chloride solution was used as the electrolyte, with a platinum sheet acting as the counter electrode and a saturated calomel electrode (SCE) as the reference electrode. The prepared sample (12.5 wt%) was mixed with paraffin, then pressed into rings of 3.0 mm inner diameter and 7.0 mm outer diameter and measured on an R & SZNB-40 vector network analyzer to study the EM parameters. Based on and transmission line theory and EM parameters ($\varepsilon=\varepsilon'-j\varepsilon''$and $\mu=\mu'-j\mu''$), the reflection loss (RL) values of samples were achieved by the following equations:

$Z_{in}=Z_{0}\sqrt{\frac{\mu}{\varepsilon}}\tanh\left(j\frac{2\pi fd\sqrt{\mu\varepsilon}}{c}\right)$ (1)

$RL=20\log\left|\frac{Z_{in}-Z_{0}}{Z_{in}+Z_{0}}\right|$ (2)


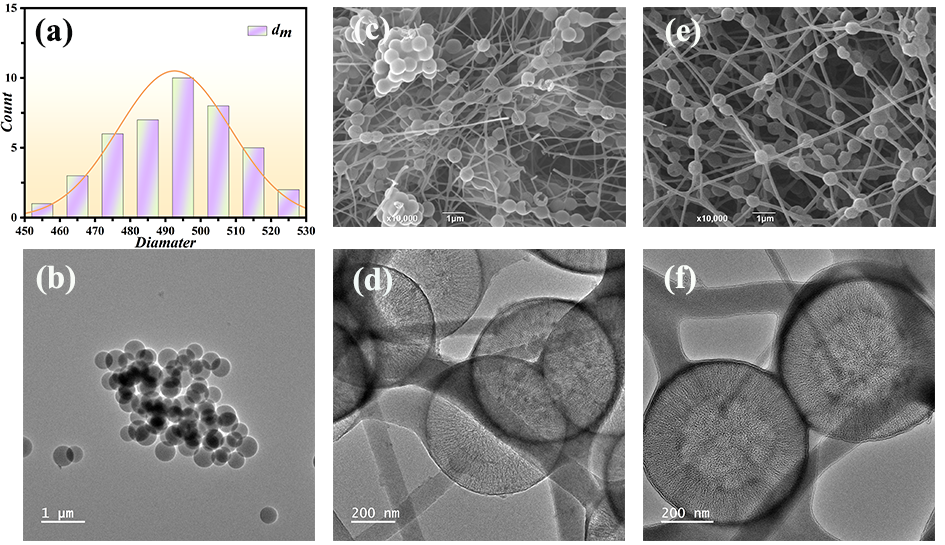


**Figure S1.** (a, b) Statistical distribution diagram spectra and TEM image of SiO_2_/carbon SNSs, (c-f) SEM and TEM of CCBNFs-2 and CCBNFs-3.


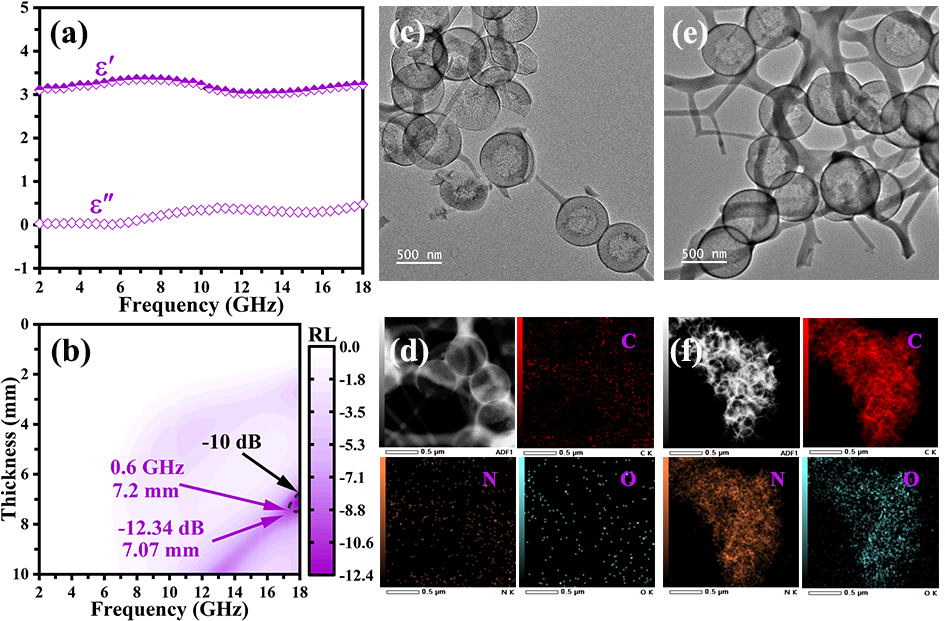


**Figure S2.** (a, b) EM parameters and RL plot of SiO₂/carbon SNSs@PAN BNFs, (c-f) TEM and element mapping images of CCBNFs-4 and CCBNFs-5.


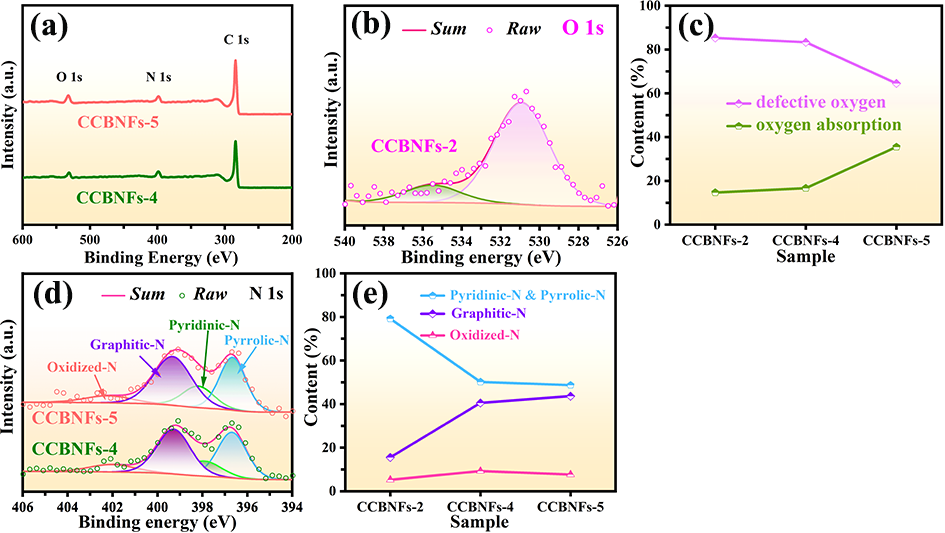


**Figure S3.** (a) XPS spectra, (b) O 1s spectra, (c) statistical chart of adsorbed oxygen and defective oxygen, (d) N 1s spectra, (e) statistical charts of pyrrole nitrogen, pyridine nitrogen, graphitic nitrogen and nitrogen oxide for CCBNFs-2, CCBNFs-4 and CCBNFs-5.


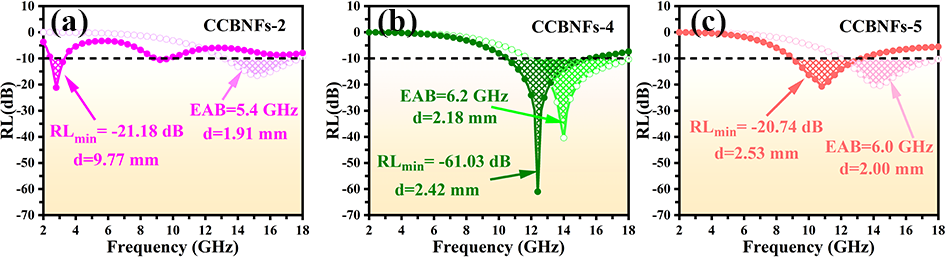


**Figure S4.** (a-c) RL curve of CCBNFs-2, CCBNFs-4, CCBNFs-5 respectively.
